# Linking transport pathways and phosphorus distribution in a loamy soil: a case study from a North-Eastern German Stagnosol

**DOI:** 10.1007/s10661-023-11465-6

**Published:** 2023-07-12

**Authors:** Stefan Koch, Henrike Lederer, Petra Kahle, Bernd Lennartz

**Affiliations:** Faculty of Agricultural and Environmental Sciences, Chair for Soil Physics, University of Rostock, Justus-Von-Liebig-Weg 6, 18059 Rostock, Germany

**Keywords:** Dye tracer experiment, Germany, Macropore flow, Mecklenburg-Vorpommern, Non-uniform flow, Preferential flow, Stained flow path width

## Abstract

Heterogeneous flow pathways through the soil determine the transport of dissolved and particle-bound nutritional elements like phosphorus (P) to ground and surface waters. This study was designed to understand the spatial patterns of P in agriculturally used soils and the mechanisms causing P accumulation and depletion at the centimetre scale. We conducted dye tracer experiments using Brilliant Blue on a loamy Stagnosol in North-Eastern-Germany. The plant-available P was analysed using double lactate extraction (DL-P). The plant-available P content of the topsoil was significantly higher than that of the subsoil in all three replicates (*p* < 0.001). The topsoil’s stained areas showed significantly higher P contents than unstained areas (*p* < 0.05), while the opposite was found for the subsoil. The P content varied enormously across all observed soil profiles (4 to 112 mg P kg^−1^ soil) and different categories of flow patterns (matrix flow, flow fingers, macropore flow, and no visible transport pathways). The P contents of these transport pathways differed significantly and followed the order: P_matrix flow_ > P_finger flow_ > P_no visible transport pathways_ > P_macropore flow_. We conclude that P tends to accumulate along flow pathways in the topsoil in the observed fertilized and tilled mineral soil. In contrast, in the subsoil at a generally lower P level, P is depleted from the prominent macroporous flow domains.

## Introduction

Phosphorus (P) is a major cause of eutrophication and surface water quality impairment. The agriculturally based non-point source pollution of water bodies is gaining more and more importance with the diminishing of point sources and the upgrade of sewage systems (Jarvie et al., 2010; Kleinman et al., 2015a; Sharpley et al., 1994). For a long time, it was the general opinion that P, because of its strong affinity to soil particles, is mainly transported at the soil surface in an erosive manner (Daniel et al., 1994). Today, we know that the rapid transport of P along preferential flow pathways vertically through the soil profile is an environmental threat, especially at tile-drained field sites where shallow ground and surface waters are directly connected (King et al., 2015, 2018; Nielsen et al., 2015; Tiemeyer et al., 2009; Williams et al., 2016). Preferential flow pathways may contribute to an elevated loss of P, especially after applying organic fertilizers with high concentrations of dissolved reactive P and after severe rainfall events (Shipitalo & Gibbs, 2005; Koch et al., 2019b). The mitigation of the diffuse P export from agricultural fields and an improved P use efficiency are critical future challenges for farmers and environmental agencies (Kleinman et al., 2015b).

Water movement in soils regulates the transport of dissolved and particle-bound nutrients and contaminants (Rozemeijer et al., 2016). Solute transport takes place through the soil matrix (e.g. uniform transport) or along preferred pathways (e.g. preferential transport (non-uniform transport)), depending on soil texture and structure. Also, hydrological factors like rainfall intensity, duration, and soil moisture affect water and solute transport.

In the case of a preferential transport situation, water flow bypasses the main parts of the porous soil matrix. This process might promote the fast transport of solutes through the vadose zone to the groundwater and, thus, can reduce the possibility of transformation and fixation processes (Gerke, 2006; Hendrickx & Flury, 2001). Likewise, bypass flow reduces the time for the desorption of P from the soil. However, the term “preferential flow” does not distinguish between the causes of non-uniform flow nor the types of flow patterns (Hendrickx & Flury, 2001). Thus, several separate terms were introduced to better describe the flow and transport situation. For instance, macropore flow occurs along continuous earthworm burrows, root channels, fissures, or cracks. It is mainly found in clayey/loamy soils where the fine texture promotes structural elements such as peds and aggregates (Wang et al., 2018). Finger flow refers to wetting columns in “finger” shape following the planar matrix wetting front. Flow fingers are not bound to an explicit soil structure and occur in loamy and sandy soils alike (Ritsema et al., 1997).

Tillage of agriculturally used soils substantially contributes to the destruction of soil structure and stable and continuous preferential flow pathways through the soil profile. While repeated tillage may destroy the persistence of the topsoil’s structure, disconnecting preferential pathways between top and subsoil (Andreini & Steenhuis, 1990), no-till or minimum-till soil treatments, as found in forests (Backnäs et al., 2012; Bol et al., 2016; Julich et al., 2017a) and grasslands (Stamm et al., 1998), allow a bio-assisted and physicochemical formation of continuous preferential flow domains. Contrary, there is an indication that ploughing may increase the risk of particle-facilitated transport under certain conditions (Schelde et al., 2006). However, soil cracks and preferential flow pathways may return within a few weeks. Furthermore, even in tilled soils, preferential flow pathways can occur under persistent or intense rain (Jarvis, 2007).

Dye tracer studies are commonly used to visualize water flow patterns in unsaturated soil profiles (Germán-Heins & Flury, 2000; Kasteel et al., 2005; Koch et al., 2016; Nobles et al., 2010). Brilliant Blue, a soluble, non-toxic tracer with high visibility, depicts uniform and non-uniform flow patterns, although it can be adsorbed to the soil with increasing clay content (Germán-Heins & Flury, 2000) and allows for the differentiation between various flow pathways and situations.

Understanding P transport across soil profiles is crucial for mitigating P losses from arable land. Soil P shows a spatial pattern, with higher concentrations in the plow layer and maximum accumulation at the surface, particularly in fertilized soils (Owens et al., 2008). The vertical decrease in P content varies depending on soil type and P fraction, with fine-textured soils accumulating non- and moderately soluble P fractions from fertilizer (Vu et al., 2009). Geostatistical studies confirm the variable spatial distribution of P in soils, both vertically and horizontally (Heilmann et al., 2005; Page et al., 2005; Wilson et al., 2016).

A depiction of the soil P distribution mainly depends on a precise P determination method. Various methods are available to detect the mobile P in soils, and comparing the different approaches remains challenging (Kruse et al., 2015). In Austria and Germany, double-lactate extraction (DL-P) and calcium-acetate-lactate extraction (CAL-P) are the main methods to determine plant-available P in soils (Schüller, 1969; VDLUFA, 1991).

**Fig. 1:**
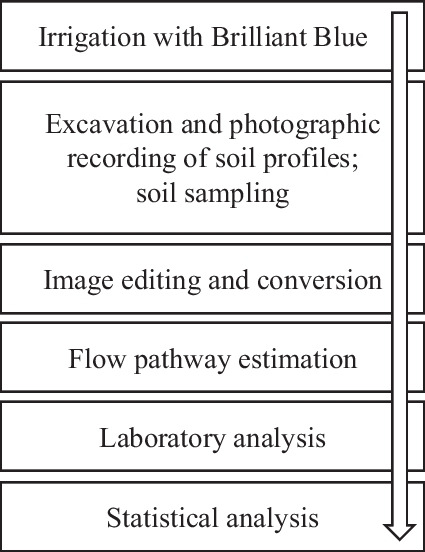
Methodological approach of the present study

In this study, we employed the dye tracer technique with Brilliant Blue (Fig. 1) to visualize the spatial patterns of water flow in tile-drained agricultural used soils in North-Eastern Germany. The study aimed to demonstrate and visualize the spatial distribution of P in soils and possibly relates soil P content to flow and transport pathways, allowing the estimation of P leaching potential to ground and adjacent surface waters.

## Materials and methods

### Study site

**Fig. 2:**
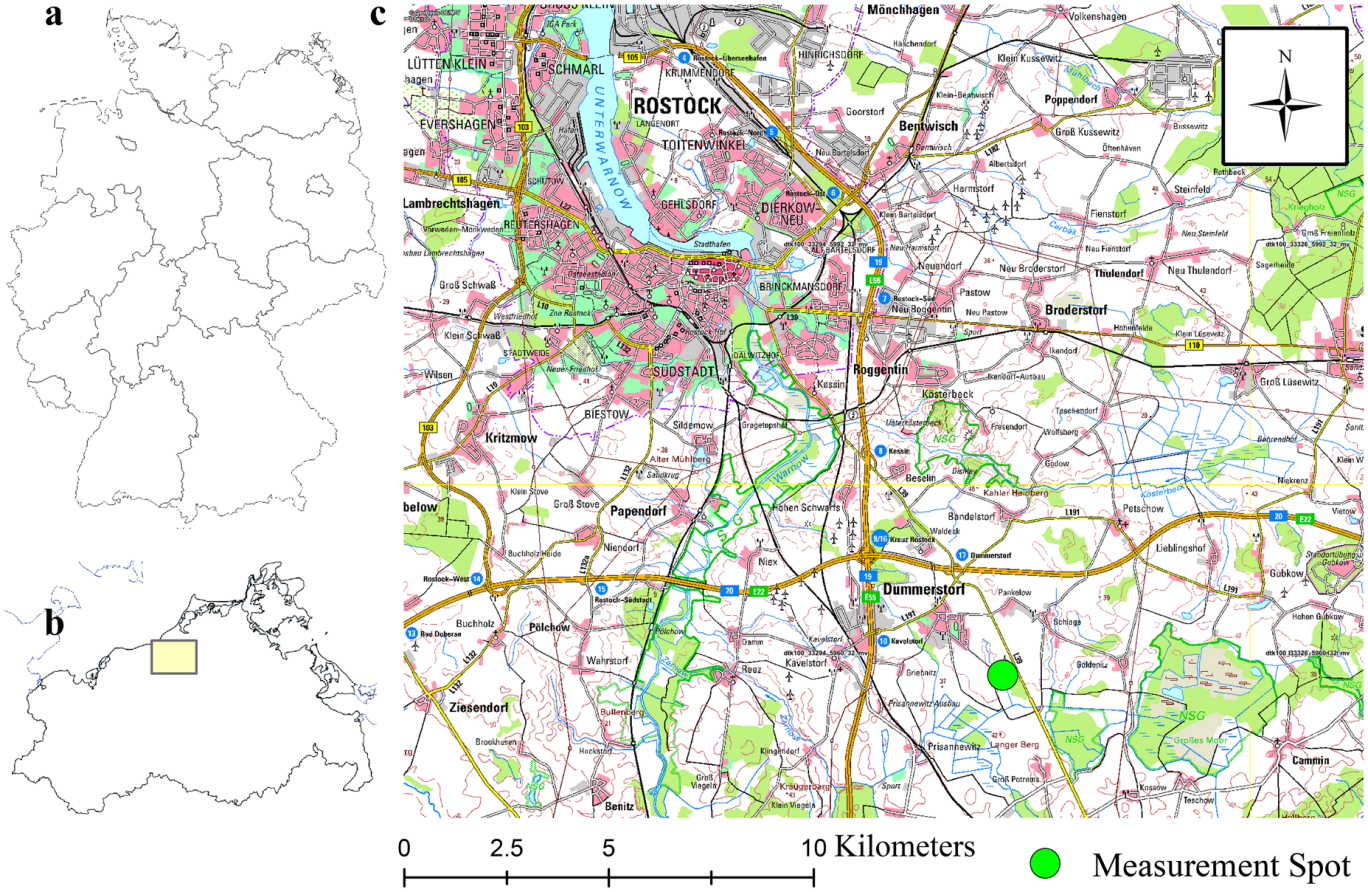
Study site in Germany (**a**), the federal state of Meckelnburg West-Pomerania (**b**), and southeast of the city of Rostock (**c**)

The study site is located 20 km southeast of the city of Rostock (Fig. 2), the administrative centre in North-Eastern Germany close to the Baltic Sea (54°00′15″ N, 12°14′45″ E). It is a Pleistocene lowland landscape with flat terrain and slight hillslopes. The landscape is predominantly agriculturally used (main crops: maize, winter wheat, winter barley, sugar beets, and winter rape), systematically artificially drained, and the human-made environment mirrors the long tradition of agriculture in North-Eastern Germany. The study area’s soils are plowed annually (0.3 m), with occasional deep ploughing (0.42 m) every 5 years. The Phacelia-covered study field was conventionally ploughed 6 months before the experiment. In addition, the study site is fertilized annually, using biogas digestate mixed with slurry with a P fertilization rate of 18 kg P ha^−1^ a^−1^. The mean DL-P contents were 80, 29, and 20 mg kg^−1^ soil for the horizons of 0–0.3 m, 0.3–0.42 m, and below 0.42 m, respectively (see horizons in Table 1).

**Table 1:** Selected soil properties (± standard deviation) at the study site, including particle size distribution (clay, silt, sand), pore size distribution (micropores, mesopores, and macropores), bulk density, carbon content (*n* = 5 per horizon). The data is derived from Koch et al. (2019a)

Depth [m]	Clay (≤ 2 μm) [%]	Silt (2–63 μm) [%]	Sand (63–2000 μm) [%]	Micropores (≤ 0.2 μm) [Vol-%]	Mesopores (0.2–0 μm) [Vol-%]	Macropores (> 50 μm) [Vol-%]	Bulk density [g cm^−3^]	Carbon content [%]
0.00–0.30	8.4 ± 0.6	28.8 ± 0.7	62.8 ± 0.2	7.6 ± 1.8	24.9 ± 4.3	13.5 ± 2.1	1.31 ± 0.3	1.4 ± 0.1
0.30–0.42	8.4 ± 0.9	28.5 ± 0.7	63.2 ± 0.4	7.6 ± 0.1	20.7 ± 0.5	12.4 ± 1.0	1.57 ± 0.02	0.6 ± 0.1
0.42–1.00	12.9 ± 1.8	31.3 ± 3.1	56.5 ± 2.5	11.5 ± 0.1	18.3 ± 1.0	3.5 ± 0.6	1.77 ± 0.02	0.7 ± 0.1

Dye tracing and soil sampling were conducted on a Stagnosol (loamy sand), according to FAO (IUSS Working Group WRB, 2015; Table 1).

### Dye tracer experiments, soil sampling, and experimental design

Three pseudo-replicate dye tracer experiments (repetitions 1, 2, and 3 with a distance of 1 m to each other) using Brilliant Blue FCF, commonly applied as a food dye and often used in soil dye tracing experiments (Forrer et al., 2000; Kasteel et al., 2013; Koch et al., 2016), were conducted. No management operations or irrigation were applied before the experiment. A 0.7 m × 0.7 m metal collar was gently pushed into the soil and covered with plastic foil. A volume of 24.5 l (corresponding to 50 mm of precipitation of Brilliant Blue solution (as suggested by Koch et al. (2016)) with a concentration of 4 g l^−1^ (Flury et al., 1994a; Gimbel et al., 2016; Koch et al., 2016) was carefully poured onto the plastic foil to prevent any soil disturbance and to ensure a uniform distribution of the water in the infiltration frame which is in accordance with Janssen and Lennartz (2008) and Liu and Lennartz (2015). Finally, the foil was gradually removed from the collar from one side to the other. Fifty millimetre is, in fact, not a regular rainfall event. However, the aim was to identify all possible preferential pathways in a well-structured loamy soil down to a depth of 70 cm. The inundation with an application of 50 mm of Brilliant Blue solution is more likely to provoke a preferential flow scenario than constant irrigation with a low application rate. This is especially the case for structured soils (Flury et al., 1994a). At flooding application, Brilliant Blue is more likely to bypass the matrix of fine-textured soil that tend to adsorb Brilliant Blue (Kasteel et al., 2002; Perillo et al., 1998, 1999).

**Fig. 3:**
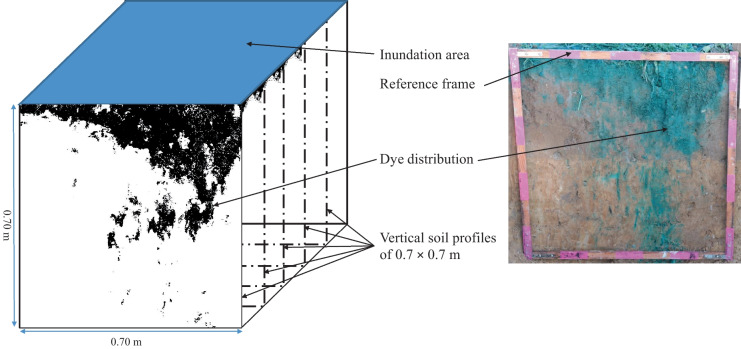
Visualization of the experimental setup of dye tracing experiments and soil sampling

Twenty-four hours after the application of the Brilliant Blue, the collars were removed from the soil, and five vertical soil profiles were excavated with a spade for each infiltration plot. The profiles were 0.7 m wide and 0.7 m deep. Five parallel soil profiles were prepared every 0.1 m across the infiltration area (Fig. 3), and each soil profile was carefully cut off with a spade. All profiles were carefully prepared with a knife and a small scoop to obtain a planar surface. In conjunction with organic compounds, clay and silt maintained a soil structure that allowed for stable soil profiles (Fig. 3).

Each profile was photographed with a digital single-lens reflex camera (Canon EOS 90D) for further image processing. For spatial referencing, a 0.7 m × 0.7 m frame with 0.1 m tick marks was pressed into the profile wall.

In each profile, soil samples were taken preferentially to cover all observed flux patterns. In total, 152 disturbed soil samples were collected for DL-P analysis by carefully cutting the soil from the profile wall with a knife and small scoops. The sampled material was carefully rasped with a razor in the shallow preferential flow pathways.

### Laboratory analysis

We assume that the plant-available P is the most significant fraction of the mobile P phase. The estimation of plant-available P in Germany is commonly done using the double-lactate extraction method (DL-P) (VDLUFA, 1991).

The P was extracted from 12 g disturbed and milled soil with 150 ml lactate solution. Concentrations of total P in the extracted solution were determined with inductively coupled plasma-optical emission spectroscopy (ICP-OES 6000 series, Thermo Fisher, Waltham, MA; USA).

### Image processing and statistical analysis

All profile images were taken in the field using grey cards and post-processed using Adobe Photoshop CC 2019 (Adobe Systems Inc.). First, the photographs were rectified (spatially corrected). Subsequently, chromatic aberration and lens distortions were corrected using Adobe Lightroom CC (Adobe Systems Inc.).

**Fig. 4:**
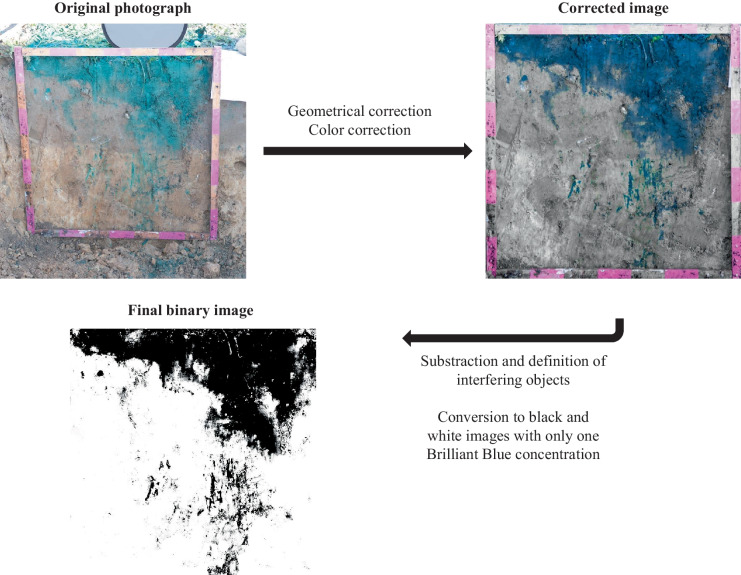
Flow chart showing the steps of image processing

Then, the images were edited manually, adjusting brightness and contrast with the preservation of a constant white balance throughout all images (Julich et al., 2017b; Koch et al., 2016; Nobles et al., 2010)(Fig. 4). Finally, colour range sampling isolated all stained areas from the unstained areas. The images were converted to images with a 700 × 700-pixel width. Hence, each pixel had an edge length of 1 mm. The final images were processed as binary black-and-white images. For further statistical evaluation, the binary images were imported into the statistical software R (R Core Development Team, 2019). Mean dye tracer coverages were calculated pixel-wise over all five prepared vertical soil profiles and all three repetitions.

**Fig. 5:**
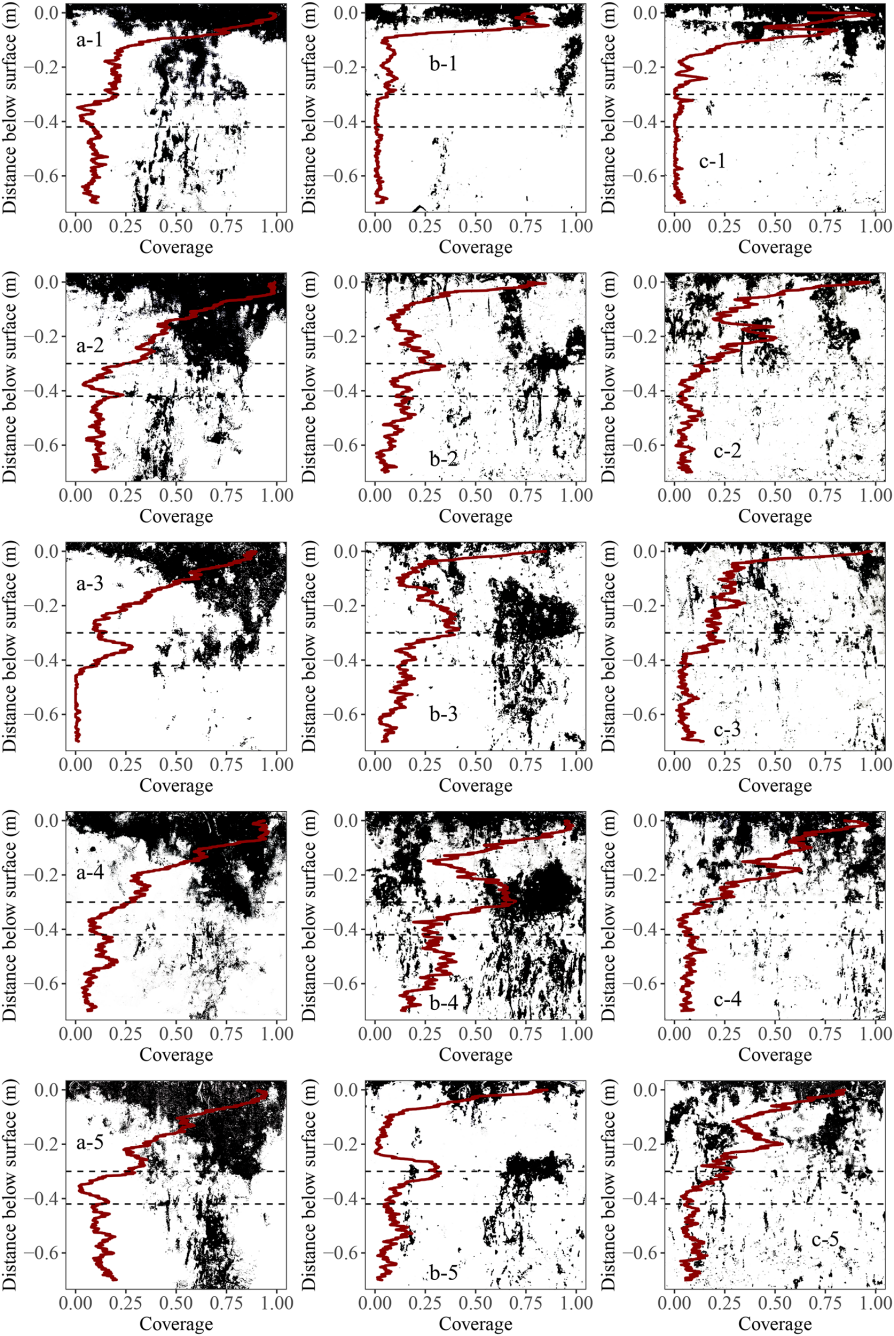
Binary images of the stained soil profiles from repetitions 1 (a-1 to a-5), 2 (b-1 to b-5), and 3 (c-1 to c-5) combined with a line plot representing the according total coverage. The profiles are 10 cm apart, from 0.05 to 0.45 m for each repetition. The entire photographic observation area is shown per profile (0.7 × 0.7 m). The horizontal lines indicate the horizon borders within the soil profile (compare Table 1)

**Fig. 6:**
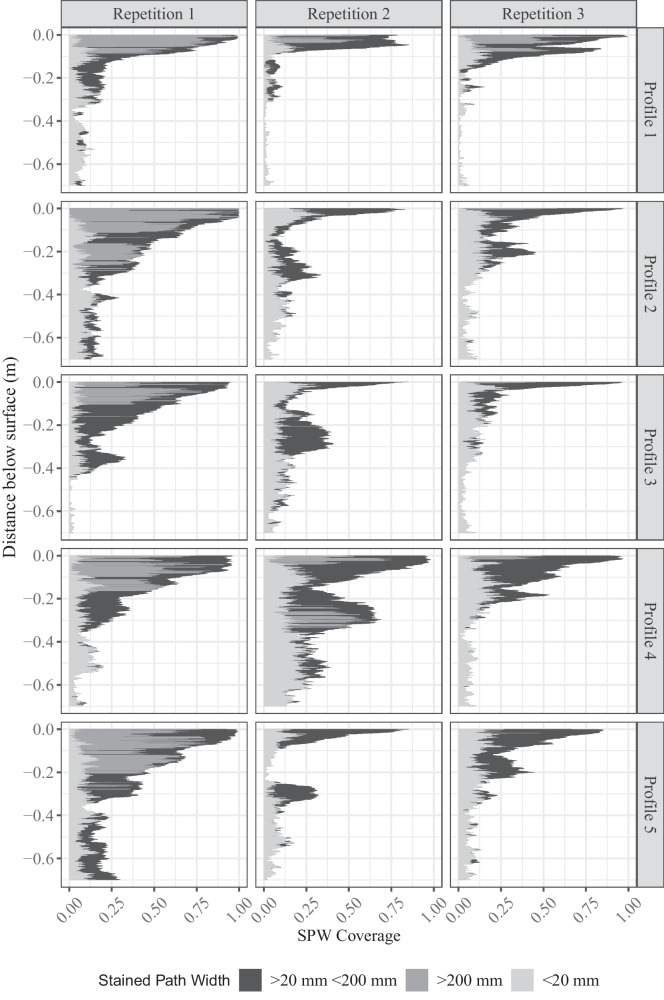
Stained path width for binary images of all stained soil profiles from repetitions 1, 2, and 3. The sum of all paths is the total dye coverage of each profile

A matrix algorithm was applied to filter and remove objects (stained areas) smaller than 2 × 2 pixels post-classification to reduce artefacts or misclassified pixels, as suggested by Julich et al. (2017b). At a pixel-wise vertical increment, the number of horizontal pixels were counted (Fig. 5) to calculate the stained path width, as Weiler and Flühler (2004) recommended (Fig. 6). The density distribution of the stained path width could be categorized as matrix flow, finger flow, macropore flow, and areas of no visible transport pathways. According to the recommendation, three flow classes based on stained path width of < 20 mm, 20 mm to 200 mm, and > 200 mm were identified: (1) *preferential flow* (with low and high interaction with the surrounding soil matrix), (2) heterogeneous matrix flow (referred to as *finger flow*), and (3) homogeneous *matrix flow and zones* not dyed by any tracer were classified as areas of *no visible flow* which was also recommended by Gimbel et al. (2016). The number of observed flow classes presented by Weiler and Flühler (2004) was reduced to (homogeneous) matrix flow, finger flow (heterogeneous matrix flow), and preferential flow to simplify the field site sampling.

## Results and discussion

### Dye tracer experiments and flow pathways

Brilliant Blue dyed marked areas of the topsoil in all replicates (Fig. 5). However, the depths of homogenous coverage as caused by matrix flow and the manifestation of flow fingers differed markedly between the three repetitions (Fig. 5). Repetition 1 exhibits the most considerable expression of matrix flow in all profiles (Fig. 3a-1 to a-5). Repetition 2 showed a severe patch of preferential flow at the border of the ploughing/deep ploughing layer through the whole soil profile (Fig. 5b-1 to b-5). Repetition 3 showed a widespread extent of macroporous pathways through the entire soil profile (Fig. 5c-1 to c-5).

The topsoil’s (0–0.30 m) mean dye tracer coverages were 50, 32, and 38% for repetitions 1, 2, and 3, respectively (Fig. 5). The corresponding subsoil (0.30–0.70 m) mean dye tracer coverages were significantly lower with 10, 13, and 6% (*p* < 0.05). In repetitions 1 and 3, there were no significant differences in the dye coverages of the individual soil profiles. In these repetitions, there were also no significant differences in the coverages between the topsoil (0.00–0.30 m) and the deep ploughing layer (0.30–0.42 m).

**Fig. 7:**
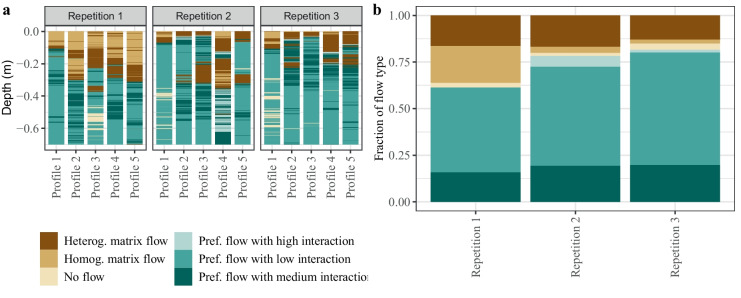
Flow pathways across all repetitions and all profiles

The quantitative analysis of the observed dye patterns confirms the qualitative description of the dye patterns. The highest occurrence of matrix flow was observed in repetition 1 (Fig. 7), where homogenous matrix flow accounted for 16% and heterogeneous matrix flow for 20% of the overall soil profile. Homogenous matrix flow was significantly more frequent than in repetitions 2 and 3 (*p* < 0.001, Mann–Whitney *U* test). However, the manifestation of heterogeneous matrix flow did not differ significantly between repetitions. The same was observed for preferential flow with high, medium, and low interaction. Preferential flow with low interaction was the most prominent flow pathway, with 46, 53, and 60% in repetitions 1, 2, and 3, respectively. Hence, it can be stated that even though the observed dye patterns show very distinct manifestations, the occurrence of the different flow pathways is astonishingly similar across all repetitions. The reason for this is probably the mathematical limitations imposed by Weiler and Flühler (2004) on the widths of the stained paths. Even though the dye patterns can vary significantly, they can still fall within the same predetermined stained path width (as shown in Fig. 6). As a result, the flow pathways depicted in Fig. 7 may appear similar. The marked finger flow pattern and the clear macropore pathways in the subsoil and the topsoil of repetitions 2 and 3 most likely originate from a pore system formed by soil aggregation and biological activity (root and earthworm channels)**.** The marked preferential flow patch at the bottom of the deep ploughing layer in repetition 2 may originate from a stable macropore system from earthworm borrows — that is not disturbed by annual ploughing (Chen et al., 2021; Dekemati et al., 2020). This transport behaviour is amplified when Brilliant Blue is applied via ponding (Flury et al., 1994b). In general, the higher clay content (12.9%, compared to below 10% in the ploughing and deep ploughing layer; see Table 1) below the ploughing and deep ploughing layer enables the formation of a stable macropore system (Bronick & Lal, 2005; Six et al., 2004), thereby facilitating the establishment of preferential flow pathways.

The individual dye patterns show a marked spatial variability of flow pathways, especially in the manifestation of preferential flow. The spatial variability of preferential flow pathways is also known from other agricultural soils with comparable particle size compositions (Grant et al., 2019; Koch et al., 2016). It is often assumed that preferential flow pathways are more likely to persist in fine-textured soils with a resilient soil structure (Grant et al., 2019; Hendrickx & Flury, 2001) as in the subsoil (0.42–0.7 m) of the present study. The deep infiltration of the dye tracer along preferential flow pathways in repetitions 2 and 3 (Fig. 5b-1 to b-5, c-1 to c5 and Fig. 6) has also been observed in soils with high clay contents of greater than 10% (Djodjic et al., 1999; Glæsner et al., 2011; Koch et al., 2016). The inundation scenario, achieved through the application of 50 mm of Brilliant Blue solution, is more likely to induce preferential flow compared to constant irrigation at low application rates, emphasizing the significance of identifying soil areas prone to preferential flow in order to understand the swift transport of nutrients and contaminants to tile drains and adjacent surface waters in agricultural soils.

### Soil P contents and their relation to the observed dye patterns

The mean DL-P contents of the topsoil (Table 2) of all profiles were 74 ± 15 mg P kg^1^ soil, which corresponds to the P content class D according to German standards (Wiesler et al., 2018). The DL-P contents of the according subsoil were significantly lower (23 ± 15 mg P kg^1^) as compared to the topsoil (*p* < 0.001). This agrees with other studies also reporting decreasing DL-P contents along the soil profile (Andersson et al., 2013; Leinweber et al., 1997; Werner et al., 2017), often characterized by a pronounced rapid decrease of P contents at the edge of the ploughing layer (Owens et al., 2008). Causally P fertilization results in a relative accumulation of P, especially in the topsoil.

**Table 2:** Mean, maximum, and minimum contents of DL-P (mg P kg^−1^ soil^−1^, *n* = 97) for all three repetitions for the topsoil and the subsoil

	Whole data set			Dyed			Undyed		
Rep	Mean	Max	Min	Mean	Max	Min	Mean	Max	min
Topsoil									
1	74	97	45	79	97	46	66	95	45
2	71	118	18	76	118	61	65	94	18
3	82	112	16	89	112	71	72	87	16
Subsoil									
1	20	47	08	17	39	08	24	47	13
2	18	50	03	14	40	03	23	50	05
3	23	68	08	22	63	10	23	68	08

The DL-P contents (mean (maximum)), taking the entire and the topsoil data set into account, were 74 (97), 71 (118), and 82 (112) (mg P kg^−1^ soil^−1^, and 79 (97), 76 (118), and 89 (112) (mg P kg^−1^ soil^−1^ for repetitions 1, 2 and 3, respectively and, hence, higher for the dyed than for the undyed soil) (Table 2). However, in the subsoil, the dyed DL-P was lower than the respective values for the unstained soil.

In the topsoil of repetitions 1 and 3, the DL-P contents differed significantly between the dyed and undyed samples (*p* < 0.05). Differences were also observed for the topsoil of repetition 2 and the subsoil of all three repetitions, albeit not statistically significant.

**Fig. 8:**
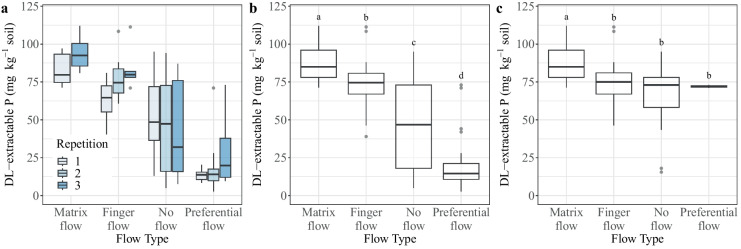
**a** Distribution of double lactate-extractable P across the entire soil profile. Repetitions are displayed separately (level of significance, *p* < 0.001; outliers are 1.5 × above and below the inner quartile range). In repetition 2, no matrix flow samples were collected. **b** Distribution of double lactate-extractable P across all repetitions and soil profiles (level of significance, *p* < 0.001; outliers are 1.5 × above and below the inner quartile range). **c** Distribution of double lactate-extractable P across all repetitions and soil profiles in the topsoil. Letters indicate significant differences in means based on a *t*-test (level of significance, *p* < 0.05; outliers are 1.5 × above and below the inner quartile range)

The mean DL-P contents of matrix flow, finger flow, no visible transport pathways, and macropore flow were 87 ± 11, 74 ± 15, 46 ± 28, and 20 ± 17 mg kg^−1^ soil^−1^, respectively. The levels of significance varied in each repetition; however, all flow types still demonstrated a significant difference across all repetitions, with a level of significance of *p* < 0.001 (Fig. 8a, b). In repetition 1, all flow types differed significantly except for finger flow and areas of no visible transport pathways (Table 3). In repetition 2, solely preferential flow and areas of no visible transport pathways did not differ significantly. In repetition 3, there was no significant difference between the DL-P contents of matrix flow and finger flow.

**Table 3:** Differences of contents of double-lactate extractable phosphorus across all repetitions and all observed flow types. *ns* no significance. In repetition 2, no matrix flow samples were collected

	Repetition 1				Repetition 2				Repetition 3			
	Matrix flow	Finger flow	No flow	Preferential flow	Matrix flow	Finger flow	No flow	Preferential flow	Matrix flow	Finger flow	No flow	Preferential flow
Matrix flow	x	*p* < 0.001	*p* < 0.05	*p* < 0.001	x	x	x	x	x	ns	*p* < 0.001	*p* < 0.001
Finger flow		x	ns	*p* < 0.001		x	*p* < 0.001	*p* < 0.001		x	*p* < 0.001	*p* < 0.001
No flow			x	*p* < 0.001			x	ns			x	*p* < 0.1
Preferential flow				x				x				x

We did not only observe a vertical decrease in soil P contents but likewise a large horizontal spatial variability. The field-scale (measured in meters) variability of soil phosphorus content, both horizontally and vertically, is documented in studies such as Heilmann et al. (2005), Vaughan et al. (2007), and Wilson et al. (2016). This variability extends to larger scales, such as catchment scale (ranging from metres to kilometres) as reported in studies like Juang et al. (2002), Jing et al. (2014), and Liu et al. (2016). At a national level, the spatial variability of soil phosphorus content spans thousands of kilometres, as reported in Leopold et al. (2006) and Roger et al. (2014). The magnitude of spatial variability changes with the spatial scale considered. The P distribution at the field scale highly depends on the pedon-scale P variability, which also is the key to understanding an elevated P leaching to groundwater (Koch et al., 2019a; Tian et al., 2017). This idea is supported by a study regarding the spatial variation of soil P at the soil profile scale on multiple forested podzols (Werner et al., 2017), where a non-uniform spatial distribution of soil P, topsoil P contents at least two times higher than in the subsoil, and a patchy P distribution in the subsoil were found. We conclude that P accumulation and transport processes differ markedly between soil horizons. We emphasize the general importance of a profile-wise process understanding as suggested by Vogel et al. (2018).

In the topsoil, areas of matrix flow showed significantly higher levels of DL-P than areas of finger flow, no visible transport pathways, and preferential flow (*p* < 0.05, Fig. 8c). The accumulation of P in the matrix flow domain of the topsoil is likely due to the fact that the pores are “younger” than the persistent subsoil macropore system. The slower flow velocities of the newly developed pore system after soil treatment allow applied P to be fixed to organic compounds and clay minerals of the topsoil. Conversely, a macropore-concentrated flow in the subsoil exerts hydraulic shear forces on pore walls provoking erosion of particle-bound P and a constant release of labile P pools. However, it must be considered that not only flow properties will affect the distribution of P.

**Fig. 9:**
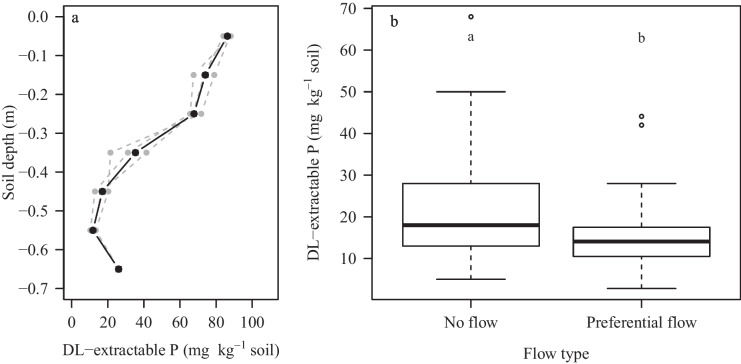
**a** Depth distribution of double lactate-extractable P. Grey lines represent mean DL-extractable P contents across all flow regions of each repetition; the black line represents the means of all repetitions. **b** Box plot of DL-extractable P contents of areas of no visible transport pathways and macropore flow in the subsoil (level of significance, *p* < 0.05; outliers are 1.5 × above and below the inner quartile range)

We observed a significant decrease in DL-P contents over depth (Fig. 9a). It is well known that in fertilized agricultural soils, a marked stratification of P can be observed with a P accumulation in the topsoil and a rapid decrease of P in the subsoil below the ploughing layer (Owens et al., 2008). It is, thus, possible that observed differences in the P distribution across the flow pathways are partially an effect of depth. However, the DL-P contents of unstained areas (no visible transport pathways) and preferential flow areas in the subsoil still differed significantly with a level of significance lower than 0.05 (Fig. 9b), indicating a depletion of P in preferential flow pathways at deeper soil depths which was also reported for German forest soils (Julich et al., 2017a; b).

Differences regarding P content between matrix flow and preferential flow pathways (finger flow and macropore flow) have been observed earlier for forest soils with persistent macro-porous structures (Julich et al., 2017a; b). Furthermore, like in the present study, no accumulation effect was found for preferential flow pathways in German forest soils (Julich et al., 2017b). We suggest that a combined effect of low P adsorption potential and high water flow rates through finger flow and macropore flow pathways cause heterogenous P distributions in sub soils. This is supported by Jensen et al. (2002) who found that the P adsorption potential of earthworm burrow soil is low as compared to bulk soil. Hence, macropore flow pathways appear to be P-depleted. The P adsorption potential is higher in soil regions with slower flow velocities (e.g. matrix flow dominated areas as formed by regular soil mixing, primarily found in the topsoil), and the soil P content exceeds the one of preferential flow pathways. Contrary, there is evidence that bio pores in the subsoil may function as a source for labile P fractions compared to the surrounding bulk soil (Bauke et al., 2017). This was not confirmed by Julich et al. (2017a) In any case, it has to be considered that topsoil P contents are generally higher in fertilized soils, as stated earlier.

The formation of preferential flow pathways, especially of macroporous flux domains, is soil structure-dependent, as is the P leaching risk. Soil structure itself and the formation of soil aggregates and macropores are linked to soil management practice such as tillage. Preferential flow pathways like earthworm burrows or root channels may, however, (re-)establish quickly and reconnect tile drains to the soil surface under any tillage practice (Nielsen et al., 2015) increasing the P leaching potential. This may lead to the assumption that periodical ploughing reduces the leaching risk by destroying the connectivity of macropores rather than no-till soil management. However, ploughing increases microbial activity in the topsoil and the concentration of P in the porewater, while no-till operations have a marked impact on organic matter, water retention, and soil compaction leading to reduced particulate P losses while the release of soluble P decreases (Daryanto et al., 2017). Subsequently, the transport of particulate P fractions (P bound to soil particles, e.g. clay minerals) can be enhanced under regular soil treatment (Schelde et al., 2006).

We conclude that the flow-induced spatial distribution of soil P may be an essential link for understanding profile-based soil processes and the complex modelling of soil functions (Vogel et al., 2018). Furthermore, the connection between soil structure, flow pathways, and P distribution may have implications for the future mitigation of soil P losses to ground and surface waters.

## Conclusion

We used the dye tracing technique and the determination of labile soil P fractions to depict the influence of flow and transport patterns on the small-scale spatial distribution of P in a sandy loam in North-Eastern Germany. Our findings demonstrate that water movement within the soil profile has a considerable impact on the spatial distribution of soil phosphorus. The soil P contents within the various observed flow and transport domains decreased in the order P_matrix flow_ > P_finger flow_ > P _no visible transport pathways_ > P_macropore flow_. Accumulation and depletion of soil P at the centimetre scale are, thus, functions of the overall P content and the governing flux regime.

We here provide small-scale insights into the importance of macropore flow onto the dislocation of P in soils and the leaching potential to ground and surface waters. It is very likely that P in shallow ground and tile drainage water originates from the subsoil where the preferential flow domain is governing flow and transport processes and which was found to be P depleted. It is, however, less clear which soil treatment and agricultural practice lower the P leaching risk in landscapes where the vertical transport of P through the soil is the dominant pathway.

## Data Availability

Not applicable.

## Abbreviations

CAL-P: Calcium acetate-lactate extraction
DL-P: Double lactate-extractable phosphorus
P: Phosphorus
